# Prognostic significance of microvessel density and other variables in Japanese and British patients with primary invasive breast cancer

**DOI:** 10.1038/sj.bjc.6604015

**Published:** 2007-10-09

**Authors:** T Kato, G Steers, L Campo, H Roberts, R D Leek, H Turley, T Kimura, S Kameoka, T Nishikawa, M Kobayashi, A L Harris, K C Gatter, F Pezzella

**Affiliations:** 1 Department of Surgery II, School of Medicine, Tokyo Women's Medical University, 8-1 Kawadacho, Shinjuku-ku, Tokyo 162-8666, Japan; 2 Cancer Research UK Tumor Pathology Group, Nuffield Department of Clinical Laboratory Sciences, University of Oxford, John Radcliffe Hospital, Headington, Oxford OX3 9DU, UK; 3 Department of Surgical Pathology, School of Medicine, Tokyo Women's Medical University, 8-1 Kawadacho, Shinjuku-ku, Tokyo 162-8666, Japan; 4 Department of Pathology, School of Medicine, Tokyo Women ‘s Medical University, 8-1 Kawadacho, Shinjuku-ku, Tokyo 162-8666, Japan; 5 Cancer Research UK Molecular Oncology Laboratory, Institute of Molecular Medicine, John Radcliffe Hospital, Headington, Oxford OX3 9DU, UK

**Keywords:** angiogenesis, breast cancer, microvessel density, international differences

## Abstract

The purpose of this study is to investigate the associations of microvessel density (MVD) and other pathological variables with survival, and whether they accounted for survival differences between Japanese and British patients. One hundred seventy-three Japanese and 184 British patients were included in the study. British patients were significantly older (56.3±11.4 years *vs* 52.5±12.9 years; *P*<0.01) and had smaller tumours (2.2±1.3 *vs* 2.7±1.8 cm; *P*<0.01), which were more frequently oestrogen receptor positive (78.8 *vs* 57.2%, *P*<0.01), had more grade III tumours (29.9 *vs* 21.4%, *P*=0.04) and more infiltrating lobular carcinomas (13.6 *vs* 4.0%, *P*<0.01) and a higher MVD compared with Japanese patients (57.9±19.8 *vs* 53.2±18.6; *P*=0.01). However, no difference in the prevalence of lymph-node metastasis was found between them (39.1 *vs* 37.5%, *P*=0.75). Younger British patients (age <50 years) had the highest MVD compared with Japanese and older British patients (*P*<0.01). Japanese patients were proportionately more likely to receive chemotherapy than endocrine therapy (*P*<0.01). British patients had a significantly worse relapse-free survival and overall survival compared with Japanese patients, after statistical adjustment for variables (hazard ratio=2.1, 2.4, *P*<0.01, *P*<0.01, respectively), especially, in T2 stage, low MVD and older subgroup (HR: 3.6, 5.0; 3.1, 3.3; 3.2, 3.9, respectively), but only in ER negative cases (*P*=0.04, *P*=0.01, respectively). The present study shows that MVD contributes to the Japanese–British disparity in breast cancer. However, the MVD variability did not explain the survival differences between Japanese and British patients.

There have been many reports about racial disparities in breast cancer incidence and outcomes. Asian-American and Japanese patients tend to have a lower incidence of breast cancer and have a better prognosis than Caucasians (Yonemoto, 1980; Sakamoto *et al*, 1981; Natarajan *et al*, 1988; Tominaga and Kuroishi, 1995; Boyer-Chammard *et al*, 1999; Braun *et al*, 2004). Age-adjusted incidence rate for breast cancer among Japanese patients has markedly increased 1.9-fold from 1978 to 1998 (age-adjusted incidence rate: 17.9, 33.8, respectively) (The Research Group for Population-based Cancer Registration in Japan, 1999, 2003; Tamakoshi *et al*, 2005) and age-adjusted death rate has increased moderately (Tominaga and Kuroishi, 1995), but is still much lower compared with those of Caucasians (Tominaga and Kuroishi, 1995; Kawamura and Sobue, 2005). Racial differences in age-adjusted mortality rates are likely to be due to many factors, such as genetics, diet, reproductive patterns, socioeconomic status, geographic and environmental exposures, and other unidentified cultural or biological factors (Iscovich *et al*, 1989; Claus *et al*, 1990; Chaudary *et al*, 1991; Gordon *et al*, 1992; Simon and Severson, 1996; Tamakoshi *et al*, 2005). In spite of numerous studies, the reason for such disparities has not been identified.

In general, angiogenesis facilitates both tumour growth and progression and it contributes to the aggressiveness of tumours. Some studies have suggested that microvessel density (MVD) as a measure of tumour angiogenesis is an independent and highly significant prognostic factor, for both node-negative and -positive patients (Weidner *et al*, 1991; Fox *et al*, 1995; Kato *et al*, 1999; Tsutsui *et al*, 2003). However, the numbers of microvessels counted by many investigators differs between studies, which also varied in patient selection, the antibody used to detect endothelial cells, sample size, method of counting microvessels, and race. Since the number of microvessels reported by Western investigators appears to be higher than that by Japanese (Axelsson *et al*, 1995; Costello *et al*, 1995; Ogawa *et al*, 1995; Kato *et al*, 1999) and green tea, consumed many times daily by the average Japanese, inhibits vascular endothelial growth factor induction in human breast cancer cells (Sartippour *et al*, 2002), we hypothesised that differences in MVD might contribute to the Japanese and British disparities in breast cancer outcomes.

This study was undertaken to investigate the associations of MVD and other variables with survival, and whether they are associated with survival differences between Japanese and British patients.

## PATIENTS AND METHODS

Two hundred and seventeen Japanese and 219 British patients were studied, excluding patients with non-invasive, Stage IV, bilateral, male, or inflammatory cancers, to investigate the characteristics of primary operable invasive female breast cancer. They had undergone breast cancer surgery at the Tokyo Women's Medical University Hospital or the John Radcliffe Hospital, Oxford between 1991 and 1993. These hospitals are tertiary referral centres. However, cases where insufficient material remained in the tissue blocks for immunohistochemical evaluation of factor VIII-related antigen were excluded. Eleven Japanese and 14 British samples for study were identified retrospectively as having no carcinoma and paraffin-embedded tissue blocks of 33 Japanese and 21 British cases were insufficient because they have been used for other research. This left 173 cases in the Japanese group and 184 cases in British group for pathological analyses.

Follow-up was from medical records. Cause of death was analysed for breast cancer alone and cases who died from other disease was defined as censored. The date of the last note in the medical record in Tokyo was 2000, while in Oxford it was 2001. Oestrogen receptor (ER) content were determined biochemically using the dextran-coated charcoal method in Tokyo and Oxford. Tumours were classified as ER-positive if the content exceeded 5 fmol *μ*g^−1^ protein. Patients' ages were categorised as age <50 years (younger group) and age ≧50 years (older group).

### Pathological studies

The pathologic specimens from both hospitals were reviewed by the same investigator (TK) without any knowledge of the eventual clinical outcome. Conventional pathological features were observed and recorded, including lymph-node status. Operative/pathological size was used for T stage in Japanese and pathological size for British patients. Grade was determined by the method of Elston and Ellis grade (Elston and Ellis, 1991). Vascular invasion was assessed on haematoxylin and eosin (H&E) sections.

### Immunocytochemical techniques

Serial sections were prepared from representative formalin-fixed and paraffin-embedded tissue blocks from this series of breast cancers. Japanese sections were fixed in 20% formalin for 48 h, while British ones in 10% formalin for the same time. The other processing was similar in Tokyo and Oxford. Tissue samples of 5 *μ*m thick sections stained with H&E were assessed histopathologically and were used to select the maximal area of the invasive components. Immunostains for factor VIII-related antigen were performed on paraffin sections using the streptavidin–biotin–immunoperoxidase method as described previously (Kato *et al*, 1999, 2003). Briefly, formalin-fixed, paraffin-embedded sections were de-waxed in 100% Citroclear, rehydrated through graded 100% industrial methylated spirit series, and immunostaining was performed using a polyclonal antibody (von Willebrand factor, Dako, Copenhagen, Denmark) applied at 1 : 200 for 1 h at room temperature. Analysis of all slides of both Japanese and British cases for H&E and factor VIII-related antigen staining was performed by the same method and at same institute (John Radcliffe Hospital, Headington, Oxford, UK) and both clinical and pathological studies were done in Tokyo.

**Figure 1:**
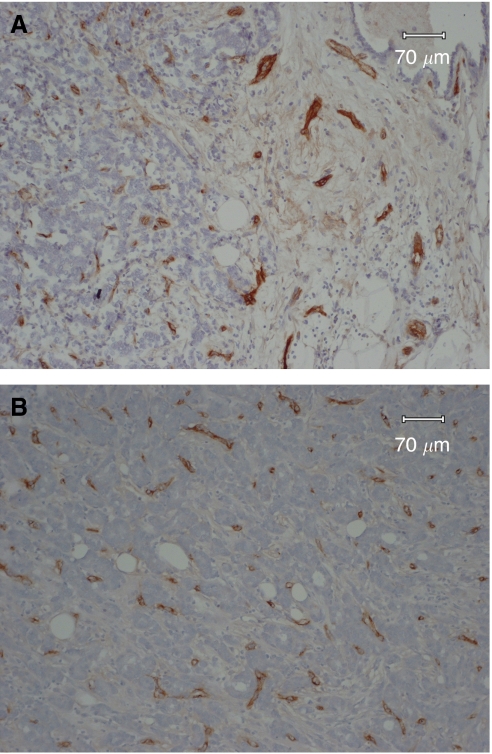
Microvessel staining: microvessels were highlighted by staining endothelial cells (staining for factor VIII-related antigen). (**A**) Example of an area from a tumour with AMC. Microvessels were highlighted by staining endothelial cells along the border between cancer nests and stroma. (**B**) Representative field of HMC showing high vascularisation.

Microvessels were counted by one investigator (TK). To evaluate the most effective method to quantify MVD in angiogenesis, two different methods were tried. The first was average microvessel count (AMC) per square millimetre (Kato *et al*, 1999, 2003). One maximal area of all the cut surfaces exhibiting invasive components in each tumour was scanned at high power (200 ×) and the number of microvessels in all the areas along the border between cancer nests and the stroma was recorded (Figure 1A). The average number of microvessels in all the fields scanned at high power was calculated giving the mean AMC. The other method was to use the three highest microvessel counts (HMC) per square millimetre (Figure 1B). These criteria were similar to those of Weidner *et al* (1991). The patients were divided into two groups (low or high) according to the median AMC or HMC value (54.4, 85.3 mm^−2^, respectively) of all patients.

### Statistical analysis

Statistical analysis of the data was performed with the Survival Tools for Statview-J 5.0 package (Abacus Concepts, Berkeley, CA, USA). All tests were two-tailed. For comparison of the median follow-up duration and the two groups and for association with T-stage, surgical treatment, ER status, lymph-node status, grade, vascular invasion and histologic type and the two groups, Mann–Whitney U-test, *χ*^2^-test or Fisher's exact tests was used. For comparison of mean age, tumour size and MVD and the two groups or four groups Mann–Whitney U-test or Kruskal–Wallis test was used. We examined the univariate relationships between prognostic indicators and relapse-free survival (RFS) and overall survival (OS) by fitting Kaplan–Meier survival curves (Kaplan and Meier, 1958) to various levels of the prognostic indicators and then looked for differences among the curves using the log–rank test (Mantel, 1966). The Cox proportional hazards regression model was also used for the multivariate analysis (Cox, 1972).

## RESULTS

### Characteristics and survival of the 436 original patient population

Two hundred and seventeen Japanese and 219 British patients underwent breast cancer surgery in Tokyo and Oxford. In the original series, British patients were significantly older (56.5±11.3 years *vs* 52.4±12.6 years, *P*<0.01) and had smaller tumours (2.1±1.6 *vs* 2.6±2.9 cm; *P*<0.01) and had more ER-positive tumours (77.2 *vs* 56.3%, *P*<0.01) compared with Japanese patients, but there was no difference in the prevalence of lymph node metastasis (37.9 *vs* 36.4%, *P*=0.74). Cox regression modelling was used to determine the effect of population and other variables on RFS and OS. British patients had a significantly worse RFS and OS compared with Japanese patients (*P*=0.01, *P*=0.01, respectively). The adjusted hazard ratio (HR) and 95% confidence intervals (CI) for RFS or OS for British patients compared with Japanese patients was 2.3 (1.4–3.5) or 2.8 (1.6–4.9) (*P*<0.01, *P*<0.01, respectively).

### Characteristics of the 357 patient subset for pathological analyses

Because of lack of tumour tissue, only a subset could be analysed for MVD. The median follow-up duration of the Japanese and British patients was 76 months (range, 1–105) and 90 months (range, 7–135) (*P*<0.01; Table 1). Clinical and pathological data are listed in Table 1. Seven patients were lost to follow-up and one patient died within 1 year. The other cases were followed up for over 1 year. Japanese patients tended to be younger, with the peak in age distribution between 40 and 49 years of age, compared to 50–59 in British patients. Mean ages were also four to five years younger in Japanese women (means 56 and 52, *P*<0.01; Table 1). Japanese patients had proportionately more T2 and T3 cases than did the British patients (46.8, 8.1 *vs* 34.8, 2.2%, respectively, *P*<0.01, Table 1). The ratio of ER-positive British patients was significantly higher than that of Japanese patients (78.8 *vs* 57.2%, *P*<0.01, Table 1). No difference in the prevalence of lymph-node metastasis was found between Japanese and British patients (39.1 *vs* 37.5%, *P*=0.75; Table 1). British patients had a higher AMC compared with Japanese patients (57.9±19.8 *vs* 53.2±18.6; *P*=0.01), but not HMC (87.9±32.3 *vs* 85.1±29.9, *P*=0.65, Table 1). Moreover, younger British patients (age <50 years) had the highest AMC compared to both younger and older Japanese and older British patients (*P*<0.01, Table 1). Patients with high AMC had proportionately more infiltrating lobular carcinoma than did the patients with low AMC (*P*=0.04). However, there was no correlation between high AMC and other variables such as T-stage, ER-positivity, age, lymph-node metastasis, high grade or vascular invasion. Japanese patients had proportionately more grade I cases than did the British patients (46.8 *vs* 34.8%, *P*=0.04, Table 1), however, there was no correlation between vascular invasion and the population (*P*=0.16). There were significant differences in the surgical treatment – for example 86.1% of Japanese patients had radical or modified radical mastectomy *vs* 17.9% of British patients (*P*<0.01), and conversely 13.9% of Japanese patients had conservative surgery (local excision alone or local excision and radiotherapy) *vs* 82.1% of British patients (*P*<0.01; Table 1). 6.5% of Japanese patients received adjuvant chemo- or chemoendocrine therapy *vs* 32.1% of British patients (*P*<0.01), and 12.1% of Japanese patients had adjuvant hormone therapy alone *vs* 62.0% of British patients (*P*<0.01). Japanese patients were proportionately more likely to receive chemotherapy than endocrine therapy.

### Relationship between prognostic variables and relapse-free survival (RFS) and overall survival (OS)

**Figure 2:**
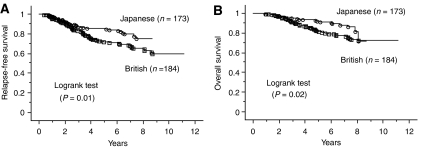
Kaplan–Meier survival curves for all patients with breast cancer. (**A**) Relapse-free survival stratified by population. (**B**) Overall survival related to population.

**Figure 3:**
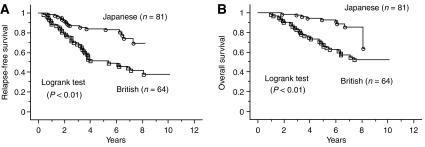
Kaplan–Meier survival curves for all patients with T2 tumours. (**A**) Relapse-free survival for the patients with T2 tumours stratified by population. (**B**) Overall survival for the patients with T2 tumours related to population.

**Figure 4:**
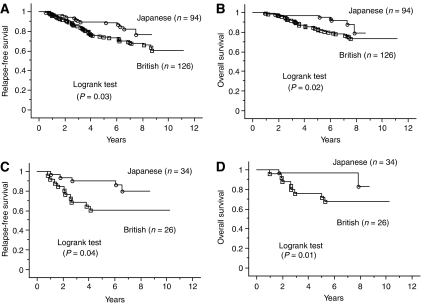
Kaplan–Meier survival curves for older patients with breast cancer. (**A**) Relapse-free survival l for all older patients stratified by population. (**B**) Overall survival for all older patients related to population. (**C**) Relapse-free survival l for ER-negative older patients stratified by population. (**D**) Overall survival for ER-negative older patients related to population.

British patients had a significantly worse RFS and OS compared with Japanese patients (*P*=0.01, *P*=0.02, respectively; Figures 2A and B). For patients in T2 stage subgroup, British patients had a significantly worse RFS and OS than Japanese patients (*P*<0.01, *P*<0.01, respectively; Figures 3A and B). Also for patients in the T3 stage subgroup, British patients had a significantly worse RFS compared with Japanese patients (*P*=0.04), but not a worse OS (*P*=0.12; curves not shown). But for patients in T1 subgroup there was no difference (*P*=0.19, *P*=0.17, respectively; curves not shown). For breast cancer stratified by ER status, ER-negative British patients had a worse OS than Japanese patients (*P*=0.04; curves not shown), but not a worse RFS (*P*=0.10). In contrast, there was no significant difference in RFS and OS amongst Japanese and British women with ER-positive cases (*P*=0.14, *P*=0.27, respectively; curves not shown). For patients in the older subgroup, British patients had a significantly worse RFS and OS than Japanese patients (*P*=0.03, *P*=0.02, respectively;Figures 4A and B) but not for patients in younger subgroup (*P*=0.15, *P*=0.50, respectively; curves not shown). Moreover, RFS and OS for ER-negative British older patients was worse than for Japanese (*P*=0.04, *P*=0.01, respectively; Figures 4C and D), while there was no significant difference in RFS and OS amongst ER-positive Japanese and British older women (*P*=0.24, *P*=0.29, respectively; curves not shown).

### Univariate and multivariate analyses of relapse-free survival (RFS) and overall survival (OS)

Model 1 of Table 2 indicates that British patients had a significantly worse RFS and OS compared with Japanese patients (HR: 2.1, 2.4, *P*<0.01, *P*<0.01, respectively) and patients with T2 or T3-stage and grade III had a significantly worse RFS and OS compared with the patients with T1-stage and grade I. However, AMC was not associated with RFS and OS in either analyses. British patients also had a significantly worse RFS and OS compared with Japanese patients in the model 2 (HR: 2.6, 3.2; *P*<0.01, *P*<0.01, respectively). In particular, British patients with T2 tumours had significantly worse RFS and OS compared with Japanese women (HR: 3.6, 5.0; *P*<0.01, *P*<0.01, respectively; Table 3). In the T-2 stage subgroup, lymph-node status was significantly associated with RFS and OS (HR: 2.4, 2.1; *P*<0.01, *P*=0.03, respectively; Table 3). Table 4 shows the unadjusted hazard ratio for age and population with multivariate adjusted hazard ratio being controlled for T-stage, nodes status, ER status, grade, AMC, and vascular invasion. The unadjusted hazard ratio for RFS or OS for older British patients compared with older Japanese patients was 1.9 or 2.4 (*P*=0.03, *P*=0.03, respectively, Table 4). After controlling for other variables, the hazard ratio of RFS or OS for them was 3.2 or 3.9 (*P*<0.01, *P*<0.01, respectively, Table 4). Older Japanese patients had better outcomes than older British patients, while there were no significant differences among younger Japanese and British patients. In the low AMC subgroup, the population and T-stag were significantly associated with RFS and OS in multivariate analysis (model 1 of Table 5). In contrast, in the high AMC subgroup, although T-stage, lymph-node status and grade significantly affected the association with RFS and OS, population had no effect on RFS and OS (model 2 of Table 5).

## DISCUSSION

Several investigators have found that Japanese patients with breast cancer have a better survival than American or British patients (Wynder *et al*, 1963; Morrison *et al*, 1976; Friedel *et al*, 1991). There are several limitations to these hospital-based studies. There is often insufficient uniformity amongst populations. There might have been a selection bias. In the previous studies, none used multivariate methods to simultaneously control the other prognostic variables. Thus, it is not known whether these survival differences were due to race or tumour variables. Our results, however, do show the better outcome in older Japanese compared with older British patients before and after adjusting for other variables (Table 4). Only other study showed that the difference in survival was restricted to postmenopausal patients (Sakamoto *et al*, 1981). Age-adjusted mortality rates in England and Wales started to decrease in the late 1980s (Beral *et al*, 1995), while those in Japan have gradually increased, but still lower than the former rates (Tominaga and Kuroishi, 1995; The Research Group for Population-based Cancer Registration in Japan, 2003) As there has been a gradual improvement in survival in British patients, the magnitude of survival differences reported in this study was not nearly as great as that reported in a previous study (Merchant *et al*, 1999). Improved survival from this cancer in the UK may be due to increased social interest, advances in diagnosis according to the NHS Breast Screening Programme, and development of adjuvant endocrine therapy and chemotherapy (Beral *et al*, 1995; Kobayashi, 2004).

There is also strong evidence to suggest a population-dependent variation in the biology of breast cancer, such as ER status, p53 gene mutation, HER-2/neu, S-phase fraction, lymphatic infiltration, sinus histiocytosis, and certain histological patterns (Sakamoto *et al*, 1981; Friedel *et al*, 1991; Elledge *et al*, 1994; Merchant *et al*, 1999). Differences in these biologic characteristics do not explain the relatively high survival rate of Japanese breast cancer patients. According to the study of Braun *et al* (2004), native Hawaiian patients had a poor survival in spite of a high ER positivity rate. The current study also showed that British patients had a high percentage of ER-positive tumours and an increased risk of death, in spite of the extensive use of adjuvant endocrine therapy. In our study the pathological variables do not explain why the survival of British patients is poorer than Japanese patients, as the former had smaller and more ER-positive tumours than the latter and the prevalence of lymph-node involvement was also the same. Therefore, we investigated MVD as a possible mechanism, because the Japanese diet is rich in antioxidants and green tea, consumed many times daily by the average Japanese and contains active antiangiogenic substances (Sartippour *et al*, 2002; Rodriguez *et al*, 2006). Thus, dietary differences involving these agents was a possible factor.

To our knowledge, this is the first study to compare the prognostic value of microvessel density in Japanese and British patients with invasive breast cancer. Various techniques for the evaluation of neovascularisation have been tested in breast cancer. Some studies have suggested that microvessel density was representative of angiogenesis of the tumour and was an independent and highly significant prognostic factor (Weidner *et al*, 1991; Horak *et al*, 1992; Gasparini *et al*, 1994; Kato *et al*, 2001; Tsutsui *et al*, 2003). However, other authors reported that angiogenesis did not predict recurrence in patients with primary breast cancer (van Hoef *et al*, 1993; Goulding *et al*, 1995). The significance of angiogenesis remains controversial due to studies which varied in patient selection, length of time of patient follow-up, antibody used to detect endothelial cells, sample size, method of counting microvessels, and the race. Some clinical studies suggested that to use an antibody to CD31 may be superior to using factor VIII-related antigen (Horak *et al*, 1992; Fox *et al*, 1995); however, another study reported that this greater sensitivity of anti-CD31 of vascular endothelium did not yield results more discriminating for predicting survival outcome than results produced with factor VIII-related antigen (Gasparini *et al*, 1994). Using an antibody to the latter antigen, many stromal vessels can be stained well as we published previously (Kato *et al*, 1999). When we compared AMC with the highest microvessel density in one or three fields (the hot spots) in the previous study, AMC was a more reliable factor than that of the hot spots (Kato *et al*, 1999). The results show that AMC was an independent prognostic factor, but its prognostic impact was not as strong as lymph-node status and clinical tumour size. The method of Weidner *et al* (1991) is popular , however, we have questioned whether or not the hot spots in one slide per tumour is representative for the whole tumour. Selection of the hot-spots area for counting is one of the possible sources of this discordance and it requires the judgment of the investigators to select which region is the most vascular (Kato *et al*, 1999, 2003; Medri *et al*, 2000). Both the Chalkley method and computerised imaging analysis may reduce the subjectivity of evaluation in quantification of microvessel count (Fox *et al*, 1995; Gasparini, 2001; Hansen *et al*, 2004). In a prior study, Kato *et al* (1999) showed that the area of highest microvessels most frequently (76.5%) appeared at the margins of the carcinoma, where invasion is taking place and active growth most likely and they counted the number of the microvessels along the border between cancer nests and the stroma (Figure 1A). The average number of microvessels in all the fields scanned at high power was calculated giving the mean AMC. We think that the method of determining AMC might be a more objective method than that of the hot spots because if the edge of the tumour for counting is decided, it is not so difficult to count the vessels, although it was somewhat subjective to determine the edge of the tumour with either ductal carcinoma *in situ* or lobular carcinoma *in situ* for counting. Thus, the significance of the methods for evaluation of microvessels remains controversial.

In the current study, to investigate the associations of microvessel density and other variables with survival, and whether they correlate with survival differences between Japanese and British patients, we used the methods of both Weidner *et al* (1991) and Kato *et al* (1999) between Japanese and British patients. British patients had a higher AMC compared with Japanese patients, but not HMC. In particular, younger British patients had the highest AMC of the 4 groups. AMC appears to be a more useful surrogate marker of tumour angiogenesis than HMC in this series. AMC and HMC were used as continuous variables at first to investigate the associations of microvessel density and other variables with survival. They were also divided into either two or three categories to analyse, but this did not significantly change the lack of association with survival. Population was significantly associated with RFS and OS in the low AMC subgroup, but had no effect on RFS and OS in high AMC subgroup. T-stage, lymph-node status and grade significantly affected the association with RFS and OS in the latter subgroup. Thus, there was no Japanese–British disparity in the more aggressive breast tumours with high AMC, tumours more than 2 cm, positive nodes or high grade. The present study shows that AMC contributes to the Japanese–British disparity in breast cancer. However, this study does not explain the favourable prognosis for Japanese patients, especially, for those who are older.

The administration of systemic adjuvant chemotherapy or endocrine therapy can improve RFS and OS in breast cancer patients (Early Breast Cancer Trialist' Collaborative Group, 1992). ER status is used to inform breast cancer treatment, and patients with ER-positive tumours are candidates for tamoxifen or similar agents. In contrast, patients with ER-negative tumours are often given chemotherapy. Boyer-Chammard *et al* (1999) reported that African-American, Hispanic, and Asian patients with breast cancer from 1984 through 1990 were more likely than Caucasian patients to receive chemotherapy and less likely to receive endocrine therapy. The current study is similar to those results. The study by the Collaborative Study Group of Adjuvant Chemoendocrine Therapy for Breast Cancer (ACETBC) in Japan was carried out from 1982 (Kasumi *et al*, 2003; Noguchi *et al*, 2005). Most of the Japanese patients in this study were treated according to that regimen. As Japanese patients had more ER-negative tumours than British patients, they were more likely to receive chemotherapy than endocrine therapy. Treatment may be a contributing factor on survival between populations. There were clear differences in the treatment policies. After controlling for population, age, AMC and vascular invasion, surgical treatment affected the association with the survival, but adjuvant treatment had no effect on the survival (Table 2). However, patients who received chemotherapy alone had significantly better prognosis than those who had no adjuvant therapy in ER-negative subgroup alone (RR: 0.1, 95% CI: 0.02–0.8, *P*=0.03).

There are several limitations to the current study. Firstly, it is not certain that the cases are comparably representative of the population cases in each country and there might have been a selection bias. However the patients came from the population local to their main cancer centres. Secondly, medical care procedures and patterns differ significantly in each country. Thirdly, there is the possibility that any systemic differences in the handling of tissue may well affect the immunohistochemical results. Fourthly, as the number of the cases we studied is less than the original selected Japanese and British cases, there may be some bias, but distribution of age, tumour size, ER status and survival in the original cases was similar to those of the cases in the pathology analysis. Finally, the length of time of follow-up varied according to the two groups, but this would not affect the survival curves for first 5 years. The advantage of this study includes consistency in the antibody used to detect endothelial cells and the methods of counting microvessels, that is same polyclonal antibody for factor VIII-related antigen was used. All slides of both Japanese and British patients for H&E and factor VIII-related antigen staining were performed according to the same method and at the same institute (John Radcliffe Hospital, Headington, Oxford, UK).

Overall therefore this study shows the difference between populations occurs in the older age group, not younger, and in the population with low AMC. Although younger British patients had the highest AMC, their prognosis was not worse than the Japanese younger patients. In the smallest tumours again there was no population difference in survival, only in the T2 stage tumours was this seen. For those with ER-positive tumours there was no difference in relapse-free or overall survival and numbers are small with this subgroup analysis. However it appears that ER-negative British patients had much worse survival than ER-negative Japanese patients. The relation to ER suggest that the growth-stimulating pathways for ER-negative cases may be different in the two populations perhaps modified by diet. Also since the difference applied to ER-negative patients, the much greater use of chemotherapy may have helped improve outcome. It will be interesting to compare a more recent series from within the last 5 years, to see if this difference has been reduced due to much greater use of chemotherapy currently in this group, ER negative over 50s, in the UK.

These findings suggest that a haematogenous pattern of dissemination may be more important than a lymphatic one for international disparities in breast cancer outcomes. It can be speculated that the other surrogate markers of tumour angiogenesis or other biological variables for haematogenous dissemination or the growth pathways in relation to ER-negative status contribute to the worse survival of British patients. Further research into the biological behaviour of international differences and their effect on the survival of patients with breast cancer should be continued.

## Figures and Tables

**Table 1:** Clinicopathological characteristics of Japanese and British patients

	**Total**			**Younger group**			**Older group**		
**Characteristics**	**Japanese (%)**	**British (%)**	***P*-value**	**Japanese (%)**	**British (%)**	***P*-value**	**Japanese (%)**	**British (%)**	***P*-value**
Patients	173	184		79	58		94	126	
*Age, years*									
Median	51	56		43	45		60	61	
Range	24–86	27–83		24–49	27–49		50–86	50–83	
Mean±s.d.	52.5±12.9	56.3±11.4	<0.01	41.3±5.8	43.3±5.2	0.03	61.9±9.0	62.3±7.9	0.48
									
*Survival follow-up, months*									
Median	76.3	90.4	<0.01	77.4	86.4	<0.01	76.6	92.9	<0.01
Range	1.2–105.7	6.9–135.8		2–101.8	14.2–127.2		1.2–105.7	6.9–135.8	
									
Recurrence	32 (18.5)	63 (34.2)	<0.01	18 (22.8)	22 (37.9)	0.05	14 (14.9)	41 (32.5)	<0.01
Deaths	20 (11.6)	45 (24.5)	<0.01	12 (15.2)	15 (25.9)	0.12	8 (8.5)	30 (23.8)	<0.01
									
Surgical treatment			<0.01			<0.01			<0.01
Radical mastectomy	149 (86.1)	33 (17.9)		70 (88.6)	11 (19.0)		79 (84.0)	22 (17.5)	
Conservative surgery	24 (13.9)	151 (82.1)		9 (11.4)	47 (81.0)		15 (16.0)	104 (82.5)	
									
Adjuvant treatment			<0.01			0.02			<0.01
Chemotherapy	55 (31.8)	15 (8.2)		29 (36.7)	13 (22.4)		26 (27.7)	2 (1.6)	
Chemoendocrine therapy	60 (34.7)	44 (23.9)		29 (36.7)	21 (36.2)		31 (33.0)	23 (18.3)	
Endocrine therapy	21 (12.1)	114 (62.0)		8 (10.1)	17 (29.3)		14 (14.9)	97 (77.0)	
None	37 (21.4)	11 (5.9)		13 (16.5)	7 (12.1)		23 (24.4)	4 (3.1)	
									
T-stage			<0.01						0.03
T1 (≦2 cm)	78 (45.1)	116 (63.0)		35 (44.3)	38 (65.5)		43 (45.7)	78 (61.9)	
T2 (>2 cm and ≦5 cm)	81 (46.8)	64 (34.8)		38 (48.1)	20 (34.5)		43 (45.7)	44 (34.9)	
T3 (>5 cm)	14 (8.1)	4 (2.2)		6 (7.6)	0		8 (8.6)	4 (3.2)	
									
*Tumour size, cm*									
Median	2.2	1.9		2.2	2		2.2	1.8	
Range	0.5–13.0	0.2–10.0		0.6–8.0	0.2–5.0		0.5–13.0	0.6–10.0	
Mean±s.d.	2.7±1.8	2.2±1.3	<0.01	2.6±1.5	2.0±0.9	0.04	2.7±2.0	2.3±1.4	0.05
									
ER status			<0.01			<0.01			<0.01
Negative	71 (42.8)	39 (21.2)		37 (49.3)	13 (22.4)		34 (37.4)	26 (20.6)	
Positive	95 (57.2)	145 (78.8)		38 (50.7)	45 (77.6)		57 (62.6)	100 (79.4)	
Unknown	7	0		4	0		3	0	
									
Lymph-node status			0.75			0.51			0.90
Negative	105 (62.5)	112 (60.9)		50 (64.1)	34 (58.6)		55 (61.1)	78 (61.9)	
Positive	63 (37.5)	72 (39.1)		28 (35.9)	24 (41.4)		35 (38.9)	48 (38.1)	
Unknown	5	0		1	0		4	0	
									
MVD									
*AMC*									
Median	50.8	57.2		51.3	64.1		50.1	64.4	
Range	12.9–107.4	4.6–132.7		18.2–107.4	26.2–132.7		12.9–100.5	4.6–108.5	
Mean±s.d.	53.2±18.6	57.9±19.8	0.01	53.4±19.7	64.0±21.0		52.9±17.7	55.0±18.7	<0.01
*HMC*									
Median	86.7	84.7		89.0	92.2		84.1	81.2	
Range	20.9–153.8	20.8–266.0		20.9–147.4	50.3–171.7		23.4–153.8	20.8–266.0	
Mean±s.d.	85.1±29.9	87.9±32.3	0.65	86.2±30.8	96.6±30.0		84.1±29.2	83.9±32.6	0.08
									
Grade			0.04			0.16			0.26
I	81 (46.8)	64 (34.8)		37 (46.8)	18 (31.0)		44 (46.8)	46 (36.5)	
II	55 (31.8)	65 (35.3)		27 (34.2)	24 (41.4)		28 (29.8)	41 (32.5)	
III	37 (21.4)	55 (29.9)		15 (19.0)	16 (27.6)		22 (23.4)	39 (31.0)	
									
Vascular invasion			0.16			0.54			0.26
Negative	114 (65.9)	125 (67.9)		45 (57.0)	36 (62.1)		69 (73.4)	89 (70.6)	
Positive	59 (34.1)	59 (32.1)		34 (43.0)	22 (37.9)		25 (26.6)	37 (29.4)	
									
Histologic type			<0.01			0.20			0.14
Infiltrating ductal carcinoma	154 (89.0)	152 (82.6)		72 (91.1)	49 (84.5)		82 (87.2)	103 (81.7)	
Infiltrating lobular carcinoma	7 (4.0)	25 (13.6)		1 (1.3)	7 (12.1)		6 (6.4)	18 (14.3)	
Others	12 (7.0)	7 (3.8)		6 (7.6)	2 (3.4)		6 (6.4)	5 (4.0)	

Younger group: age <50 years; Older group: age ≧50 years.

ER=oestrogen receptor; MVD=microvessel density; AMC=average microvessel counts; HMC=highest microvessel counts.

Younger group: age <50 years; Older group: age ≧50 years.

Per cent do not include cases of unknown ER or lymph-node status.

**Table 2:** Multivariate analysis of the value of prognostic factors for relapse-free survival and overall survival among all Japanese and British patients

**Model**	**Model 1**						**Model 2**					
	**Relapse-free survival**			**Overall survival**			**Relapse-free survival**			**Overall survival**		
**Variable**	**HR**	**95% CI**	***P-*value**	**HR**	**95% CI**	***P-*value**	**HR**	**95% CI**	***P-*value**	**HR**	**95% CI**	***P-*value**
*Population*												
British *vs* Japanese	2.1	1.3–3.6	<0.01	2.4	1.3–4.4	<0.01	2.6	1.4–4.9	<0.01	3.2	1.6–6.6	<0.01
												
*Age group*												
Younger *vs* older	1.3	0.9–2.1	0.16	1.3	0.8–2.1	0.33	1.2	0.8–1.9	0.41	1.1	0.7–1.9	0.65
												
*T-stage*												
T2 vs T1	2.5	1.6–4.0	<0.01	2.8	1.6–4.9	<0.01						
T3 vs T1	4.6	1.9–11.1	<0.01	7.1	2.8–18.0	<0.01						
												
*Lymph-node status*												
Positive *vs* negative	2.4	1.6–3.7	<0.01	2.5	1.5–4.1	<0.01						
												
*ER status*												
Negative *vs* positive	1.1	0.7–1.8	0.73	1.2	0.7–2.1	0.56						
												
*Grade*												
II *vs* I	1.9	1.1–3.2	0.01	1.9	1.0–3.6	0.05						
III *vs* I	1.9	1.1–3.5	0.03	2.2	1.1–4.5	0.02						
												
*AMC*												
High *vs* low	0.9	0.6–1.4	0.58	1.0	0.6–1.7	0.98	0.8	0.6–1.3	0.41	0.9	0.6–1.5	0.78
												
*Vascular invasion*												
Positive *vs* negative	0.9	0.6–1.3	0.48	0.8	0.5–1.4	0.47	1.1	0.8–1.8	0.51	1.1	0.7–1.9	0.63
												
*Surgical treatment*												
Mastectomy *vs* conservative							0.6	0.3–1.0	0.05	0.5	0.3–0.9	0.01
												
*Adjuvant tratment*												
Chemo alone *vs* None							2.7	0.9–8.1	0.06	1.5	0.5–4.7	0.50
Chemoendocrine *vs* None							3.0	1.1–8.4	0.03	1.8	0.6–5.2	0.28
Endocrine alone *vs* None							1.9	0.6–5.4	0.25	1.2	0.4–3.5	0.80

AMC=average microvessel counts; ER=estrogen receptor; HR=hazards ratio; 95% CI= 95% confidence interval.

Younger group: age <50 years; Older group: age ≧50 years

Multivariate model 1 adjusted for population, age, T-stage, lymph-node status, ER status, grade, AMC and vascular invasion.

Multivariate model 2 adjusted for population, age, AMC, vascular invasion, surgical treatment and adjuvant treatment.

Hazards ratio from Cox regression analysis.

**Table 3:** Multivariate analysis of the value of prognostic factors for relapse-free survival and overall survival among Japanese and British patients with T2 tumours

	**Relapse-free survival**			**Overall survival**		
**Variable**	**HR**	**95% CI**	***P-*value**	**HR**	**95% CI**	***P-*value**
*Population*						
British *vs* Japanese	3.6	1.9–6.9	<0.01	5.0	2.1–11.8	<0.01
						
*Age group*						
Younger *vs* older	1.1	0.6–1.9	0.82	1.1	0.5–2.2	0.81
						
*Lymph-node status*						
Positive *vs* negative	2.4	1.4–4.3	<0.01	2.1	1.1–4.1	0.03
						
*ER status*						
Negative *vs* positive	1.6	0.8–3.1	0.20	2.0	0.9–4.6	0.08
						
*Grade*						
II *vs* I	1.7	0.6–3.5	0.14	1.6	0.6–4.0	0.30
III *vs* I	1.2	0.5–2.8	0.65	1.5	0.6–4.1	0.42
						
*AMC*						
High *vs* low	0.7	0.4–1.3	0.24	0.8	0.4–1.6	0.45
						
*Vascular invasion*						
Positive *vs* negative	1.1	0.6–1.9	0.78	1.0	0.5–2.0	0.98

AMC=average microvessel counts; ER=oestrogen receptor; HR=hazards ratio; 95% CI= 95% confidence interval.

Younger group: age <50 years; Older group: age ≧50 years.

Multivariate models adjusted for population, age, lymph-node status, ER status, grade, AMC and vascular invasion.

Hazards ratio from Cox regression analysis.

**Table 4:** Age-specific models among British patients compared with Japanese patients

	**Relapse-free survival**			**Overall survival**		
**Variable**	**HR**	**95% CI**	***P*-value**	**HR**	**95% CI**	***P*-value**
*Older group (Age* ≧*50 years)*						
Unadjusted						
Population (British *vs* Japanese)	1.9	1.1–3.6	0.03	2.4	1.1–5.2	0.03
AMC (high *vs* low)	1.2	0.7–2.1	0.41	1.4	0.7–2.7	0.27
						
*Multivariate adjusted*						
Population (British *vs* Japanese)	3.2	1.6–6.3	<0.01	3.9	1.7–8.9	<0.01
AMC (high *vs* low)	1.3	0.7–2.3	0.35	1.3	0.7–2.5	0.46
						
*Younger group (Age* <*50 years)*						
* Unadjusted*						
Population (British *vs* Japanese)	1.6	0.8–2.9	0.15	1.4	0.6–3.1	0.35
AMC (high vs low)	0.6	0.3–1.1	0.12	0.8	0.4–1.7	0.53
						
* Multivariate adjusted*						
Population (British *vs* Japanese)	1.5	0.7–3.2	0.29	1.1	0.4–2.8	0.86
AMC (high *vs* low)	0.6	0.3–1.1	0.09	0.9	0.4–2.0	0.78

AMC=average microvessel counts; ER=oestrogen receptor; HR=hazards ratio; 95% CI= 95% confidence interval.

Multivariate models adjusted for population, T-stage, lymph-node status, ER status, grade, AMC and vascular invasion.

Hazards ratio from Cox regression analysis.

**Table 5:** Multivariate analysis of the value of prognostic factors for relapse-free survival and overall survival among Japanese and British patients with low and high AMC tumours

**Model**	**Model 1 (low AMC)**						**Model 2 (high AMC)**					
	**Relapse-free survival**			**Overall survival**			**Relapse-free survival**			**Overall survival**		
**Variable**	**HR**	**95% CI**	***P-*value**	**HR**	**95% CI**	***P-*value**	**HR**	**95% CI**	***P-*value**	**HR**	**95% CI**	***P-*value**
*Population*												
British *vs* Japanese	3.1	1.5–6.4	<0.01	3.3	1.3–8.2	<0.01	1.7	0.8–3.4	0.14	1.7	0.8–3.9	0.19
												
*Age group*												
Younger *vs* older	2.3	1.2–4.3	<0.01	1.9	0.9–3.9	0.09	1.0	0.6–1.9	0.91	1.2	0.6–2.4	0.68
												
*T-stage*												
T2 vs T1	3.0	1.5–5.8	<0.01	3.9	1.7–8.8	<0.01	2.5	1.3–4.8	<0.01	2.5	1.1–5.4	0.02
T3 vs T1	1.7	0.3–8.2	0.49	3.9	0.7–20.2	0.10	22.1	6.4–76.4	<0.01	15.3	4.3–54.7	<0.01
												
*Lymph-node status*												
Positive *vs* negative	2.5	1.4–4.4	<0.01	1.9	0.9–4.0	0.06	2.1	1.1–3.9	0.02	2.9	1.3–6.3	<0.01
												
*ER status*												
Negative *vs* positive	1.0	0.5–1.9	0.95	1.0	0.5–2.4	0.91	1.5	0.7–3.1	0.26	1.8	0.8–4.2	0.17
												
*Grade*												
II *vs* I	1.5	0.7–3.1	0.25	1.4	0.6–3.3	0.48	2.8	1.3–6.2	0.01	3.1	1.2–8.4	0.02
III *vs* I	1.5	0.6–3.4	0.36	1.5	0.6–4.0	0.38	3.9	1.6–9.4	<0.01	4.2	1.4–12.9	0.01
												
*Vascular invasion*												
Positive *vs* negative	1.1	0.6–1.9	0.78	0.9	0.4–1.9	0.73	0.6	0.3–1.2	0.12	0.8	0.3–1.7	0.49

HR=hazards ratio; 95%CI=95% confidence interval.

ER=oestrogen receptor; AMC=average microvessel counts.

Younger group: age <50 years; Older group: age ≧50 years.

Multivariate models adjusted for population, age, T-stage, lymph-node status, ER status, grade and vascular invasion.

Hazards ratio from Cox regression analysis.
